# KRAS Mutation Analysis by PCR: A Comparison of Two Methods

**DOI:** 10.1371/journal.pone.0115672

**Published:** 2015-01-08

**Authors:** Louise Bolton, Anne Reiman, Katie Lucas, Judith Timms, Ian A. Cree

**Affiliations:** 1 Department of Pathology, Queen Alexandra Hospital, Portsmouth, United Kingdom; 2 Department of Pathology and Warwick Medical School, University Hospitals Coventry and Warwickshire, Coventry, United Kingdom; CEA - Institut de Genomique, FRANCE

## Abstract

**Background:**

KRAS mutation assays are important companion diagnostic tests to guide anti-EGFR antibody treatment of metastatic colorectal cancer. Direct comparison of newer diagnostic methods with existing methods is an important part of validation of any new technique. In this this study, we have compared the Therascreen (Qiagen) ARMS assay with Competitive Allele-Specific TaqMan PCR (castPCR, Life Technologies) to determine equivalence for KRAS mutation analysis.

**Methods:**

DNA was extracted by Maxwell (Promega) from 99 colorectal cancers. The ARMS-based Therascreen and a customized castPCR assay were performed according to the manufacturer’s instructions. All assays were performed on either an Applied Biosystems 7500 Fast Dx or a ViiA7 real-time PCR machine (both from Life Technologies). The data were collected and discrepant results re-tested with newly extracted DNA from the same blocks in both assay types.

**Results:**

Of the 99 tumors included, Therascreen showed 62 tumors to be wild-type (WT) for KRAS, while 37 had KRAS mutations on initial testing. CastPCR showed 61 tumors to be wild-type (WT) for KRAS, while 38 had KRAS mutations. Thirteen tumors showed BRAF mutation in castPCR and in one of these there was also a KRAS mutation. The custom castPCR plate included several other KRAS mutations and BRAF V600E, not included in Therascreen, explaining the higher number of mutations detected by castPCR. Re-testing of discrepant results was required in three tumors, all of which then achieved concordance for KRAS. CastPCR assay Ct values were on average 2 cycles lower than Therascreen.

**Conclusion:**

There was excellent correlation between the two methods. Although castPCR assay shows lower Ct values than Therascreen, this is unlikely to be clinically significant.

## Introduction

**Figure 1:**
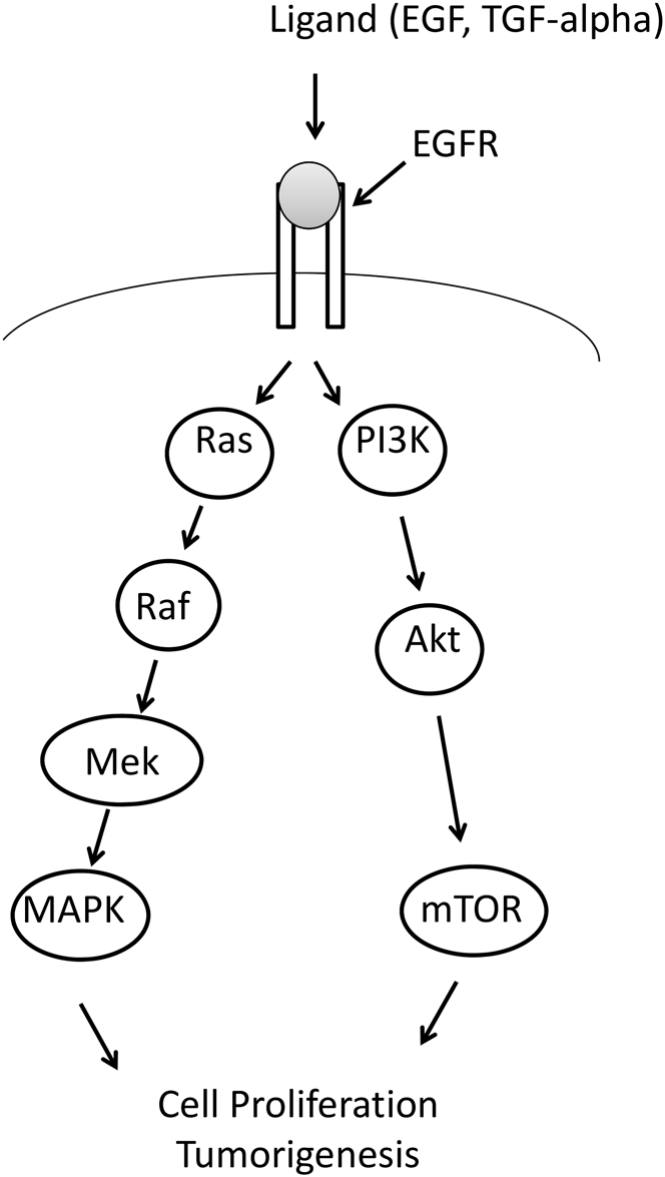
Diagram showing the contribution of KRAS and BRAF to the EGFR pathway. Activating mutations in both BRAF and KRAS will result in EGFR independent cell proliferation and hence resistance to anti-EGFR antibody treatment.

Colorectal carcinogenesis involves multiple steps with accumulation of numerous acquired genetic and epigenetic events [1, 2]. Only a small fraction of these alterations actually drive tumorigenesis initiating the transformation of normal colonic epithelium and lead to the development of malignant carcinomas and eventually advanced metastatic disease. The understanding of colorectal cancer (CRC) biology is rapidly growing and several molecular pathways including Wnt- β-catenin, TGF—β and epidermal growth factor receptor (EGFR) signalling have been identified that are deregulated at different stages of colon carcinogenesis [2, 3]. Genetic alterations in CRC show promise as potential biomarkers for early cancer diagnosis as well as in selection of patients for treatment [4–8]. In recent years targeted therapies inhibiting EGFR signalling have been introduced into clinical practice resulting in improvement of overall survival of a subset of patients with advanced metastatic disease. Mutations in the *KRAS* proto-oncogene are now widely recognized to be predictive for primary as well as acquired resistance to tailored therapy with anti-EGFR antibodies in colorectal cancer [9–11]. Approximately 40% of CRCs harbour mutations of *KRAS* that occur at early stages of the disease and are present throughout tumor progression to metastatic stages [1, 12–14]. *KRAS* mutations are usually in exon 2 with approximately 80% missense mutations in codon 12 and 20% in codon 13. The functional consequence of mutations in these two codons is activation of EGRF-Ras-Raf-MAPK-pathway (Fig. 1) that impairs the response of cancer cells to anti-EGFR antibody therapies. Likewise, rare *KRAS*-activating mutations in codons 61 and 146 [9, 15] may be also associated with attenuated response to therapy with targeted drugs [16].

*KRAS* mutation assays are important companion diagnostic tests to guide the use of anti-EGFR antibody treatment of metastatic colorectal cancer. U.S. Food and Drug Administration (FDA) and the European Medicines Evaluation Agency require that *KRAS* mutation status is determined prior to anti-EGFR treatment. As a high proportion of late stage colorectal cancers will relapse, it is common to perform these tests on the primary tumor. In many pathology laboratories the diagnosis of a cancer with high metastatic potential or which has spread triggers an automatic request for *KRAS* testing. Such ‘reflex testing’ by the pathology laboratory has the advantage that results are available immediately if metastatic disease occurs and more quickly in those cases with metastases.

The primary tumor usually has ample material for assay, in the form of formalin-fixed paraffin-embedded (FFPE) blocks, from which samples with high neoplastic cell content can be obtained [17]. Small biopsies of inoperable or metastatic disease are more challenging, and may contain few neoplastic cells. The current assays for *KRAS* genotyping in tumor samples include direct sequencing of genomic DNA and PCR based assays [18]. Although sequencing offers better coverage of the coding sequence and identifies specific locations of genetic alterations in the gene, it is relatively time consuming and its diagnostic sensitivity depends on the mutant/wild-type allele ratio present in the tumor that makes the detection of point mutations particularly challenging. PCR based methods are more sensitive but do not test for less frequently occurring mutations with as yet uncertain clinical implications. The present study compares Therascreen (Qiagen) assay with in house Taqman Mutation Detection Assays powered by castPCR technology (Life Technologies), hereafter called ‘castPCR’, to determine equivalence for *KRAS* mutation analysis. The Therascreen assay was chosen as the comparator as it was the method in use in the laboratory, and is widely used in both clinical and trial settings.

## Methods

### Samples

A total of 99 tumors were included in the study from those submitted for routine histopathology to either of the two centres involved. Inclusion criteria were a diagnosis of colorectal cancer following surgical resection, with sufficient tissue blocks taken to select one for assay. All were obtained from patients with written consent for use of tissue surplus to diagnostic requirements according to tissue bank ethics approval. Samples from Portsmouth were drawn from the Portsmouth Molecular Pathology Tissue Bank, approved by the UK National Research Ethics Service (NRES), North West Research Ethics Committee, and Coventry samples were drawn from the Arden Tissue Bank, approved by the UK NRES South Central Research Ethics Committee. There were no exclusion criteria. For each case, the histology was reviewed to confirm colorectal cancer, a block selected by the histopathologist, and areas identified on an H&E slide from which cores should be taken for KRAS mutation detection. Cores were taken using a manual tissue arrayer (MTA1; Beecher Instruments Inc., Sun Prairie, WI, USA) fitted with a punch stylet 1.0 mm in diameter was aligned over the desired area of interest (AOI), which was punched out from the block. The stylet was decontaminated (DNA Zap, Life Technologies) and cleaned (70% alcohol) between each FFPE block. In Coventry, punches were taken using a disposable 1mm skin punch with an ejection mechanism (Meditech Systems Ltd, Shaftesbury, Dorset, UK). A minimum of two 1.0 mm diameter cores were obtained from each block and placed in a sterile labelled 1.5 ml microcentrifuge tube.

### Mutational analysis of *KRAS* and *BRAF*

Genomic DNA was extracted from two cores obtained from areas of colorectal cancer (>50% neoplastic cells) identified by a pathologist in blocks of formalin-fixed paraffin-embedded (FFPE) tissue in 99 tumors. DNA extraction was performed using the automated Maxwell 16 Instrument with the Maxwell 16 FFPE Plus LEV DNA Purification Kit (Promega), following the manufacturer’s instructions. DNA content was determined by Nanodrop sphectrophotometry. Samples were subjected to mutation analysis using ARMS-based Therascreen assay and castPCR method according to manufacturer’s recommendations. In Portsmouth both assays were performed in 96-well plates, on an Applied Biosystems 7500 Fast Dx real-time PCR machine (Life Technologies), while in Coventry, the Therascreen assays was performed in tubes in a Rotorgene PCR machine, and the castPCR assays in 96 well plates in a ViiA7 PCR machine (Life Technologies). All assays were performed without knowledge of the results of the other assay by trained biomedical scientists (LB, AR and KL). The data were collected and discrepant results re-tested with newly extracted DNA from the same blocks in both assay types.

### Therascreen *KRAS* PCR

Each sample was tested for the presence of 7 KRAS mutations in codons 12 and 13 using Therascreen *KRAS* PCR Kit (Qiagen Ltd, Manchester, UK). KRAS (NM_004985) mutations G12A, G12D, G12R, G12C, G12S, G12S and G13D were included in the kit. Therascreen *KRAS* PCR is based on two systems, ARMS (Amplification Refractory Mutation System) and Scorpions [19–21] for allele specific amplification and detection of amplification, respectively. The reaction volume was 25 µl, with 80 ng of input DNA, and cycling conditions were as follows: initial denaturation at 95° for 4 min followed by 40 cycles of denaturation at 95° for 30 sec and annealing at 60°C for 1 minute. The sample ΔCt values were calculated as the difference between the Ct value of the mutation assay and the Ct value of the control assay from the same sample and samples were reported to contain a mutation if the ΔCt was sufficient according to an analysis matrix defined by the manufacturer. The control assay, labelled with FAM, is used to assess the total DNA in a sample and amplifies a region of exon 4 of the *KRAS* gene, avoiding known polymorphisms. Reported sensitivity for mutations in Therascreen is 1% (www.qiagen.com).

### Competitive Allele-Specific TaqMan PCR (CastPCR)

**Figure 2:**
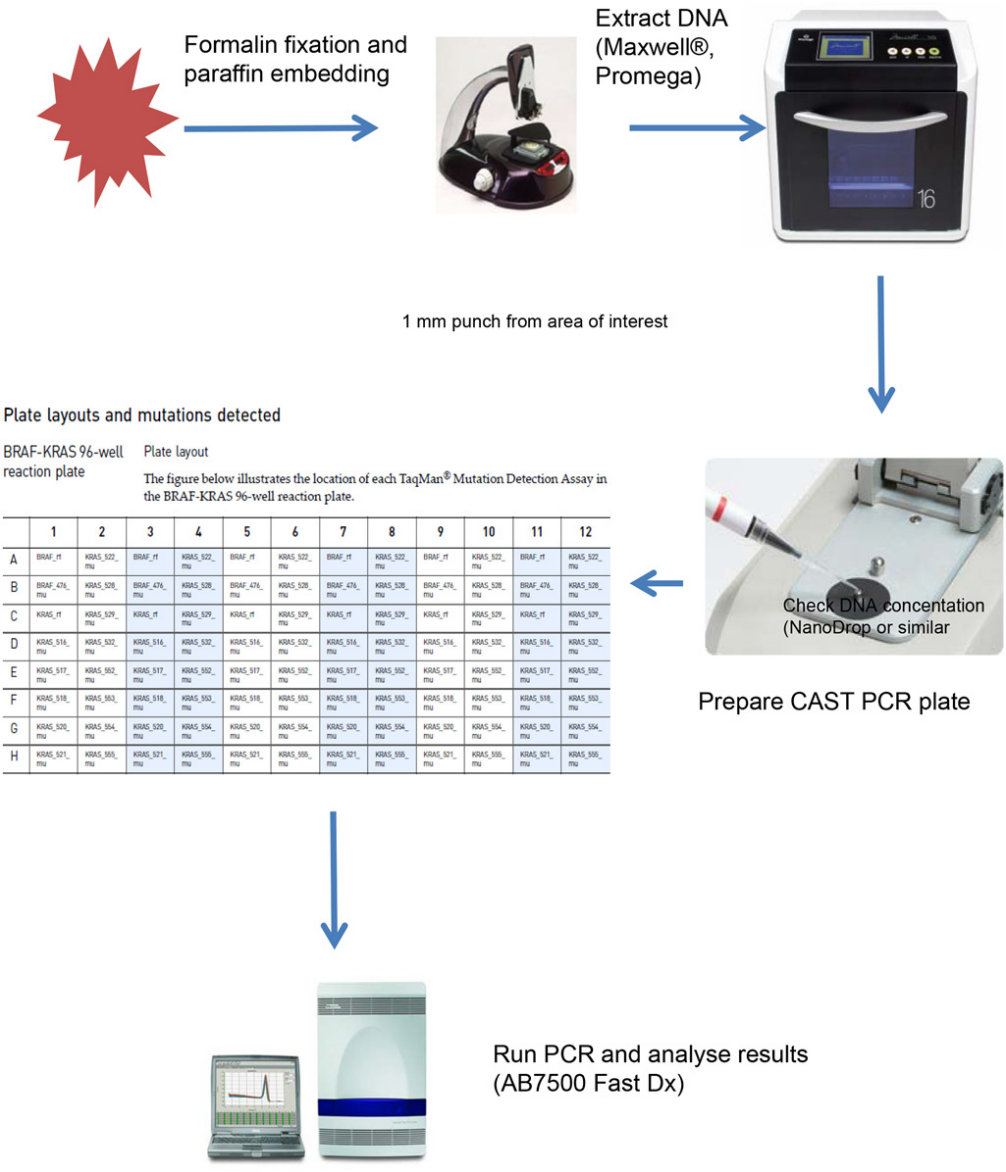
Sample flow for CAST PCR using automated extraction to allow results to be generated in <24 hours.

The sample flow of castPCR is illustrated in Fig. 2. The mutation detection assay for all 99 tumor samples was performed in 96-well plates, each containing 6 replicates of the custom BRAF-KRAS panel. Each replicate detects 1 wild type *BRAF* and 1 wild type *KRAS* gene reference sequence within exon 3 of *BRAF* and *KRAS* respectively, the *BRAF* V600E mutation and 13 distinct *KRAS* mutations (Life Technologies, Paisley, UK) as shown in Table 1. 50 ng of gDNA was used per reaction and the reaction volume was 20 µl. Cycling conditions were 95⁰ for 10 min followed by 5 cycles of 92⁰ for 15 sec and 58⁰ for 1 min and 40 cycles of 92⁰ for 15 sec and 60⁰ for 1 min. Reported sensitivity for mutations is down to 0.1% mutation in a background of wild type DNA [22].

**Table 1:** Summary of results.

**Mutation**	**Amino Acid Change**	**Cast PCR**	**Therascreen**
BRAF c.1799T>A	p.V600E	13	-
KRAS c.34G>T	p.G12C	5	5
KRAS c.34G>A	p.G12S	1 (2)	1
KRAS c.34G>C	p.G12R	3	3
KRAS c.35G>T	p.G12V	10	10
KRAS c.35G>A	p.G12D	10	10
KRAS c.35G>C	p.G12A	2 (1)	2 (3)
KRAS c.37G>A	p.G13S	0	-
KRAS c.37G>C	p.G13R	0	-
KRAS c.38G>A	p.G13D	6	6
KRAS c.182A>G	p.Q61R	1	-
KRAS c.182A>T	p.Q61L	0	-
KRAS c.183A>C	p.Q61H	0	-
KRAS c.183A>T	p.Q61H	0	-
Total of KRAS mutant		38 (38)	37 (38)
Total BRAF mutant		13	-
Total wild-type		48	62
Total Patients		99	99

Therascreen does not include Q61 mutations or *BRAF*. One tumor had *KRAS* p.G13D and p.Q61R mutations, while a further tumor had *BRAF* p.V600E and *KRAS* p.G12V mutations. This table includes re-testing results in three tumors (orginal results in brackets), and dual mutations in two tumors. One tumor was called wild-type by castPCR, and mutant by Therascreen, with a mutation by both methods on re-testing. Two further tumors were called mutant by either castPCR or Therascreen: both were wild-type on re-testing.

### Data analysis

Both tests identify assay failure or success based on reference gene Ct values, and the presence or absence of a mutation based on ΔCt values according to criteria defined by the manufacturer. The results were tabulated (Table 1) and discordant results identified. These tumors were re-tested by both assays using newly extracted DNA from further cores from the same or an adjacent tissue block from the tumor. Statistically, sensitivity was calculated as the proportion of tumors with *KRAS* mutation identified by castPCR/proportion of tumors with *KRAS* mutation identified by Therascreen, ignoring those mutations present in castPCR that are not tested by Therascreen. Specificity was defined as the proportion of wild-type tumors identified by castPCR/proportion of wild-type tumors identified by Therascreen. The primary measure of concordance (p) was also determined, as used by Chang *et al.* [23] as the proportion of the *KRAS* wild-type (Therascreen assay) patients who were also identified as KRAS wild-type by the comparator: p = [a/(a + b)]. An alternative method is to use Cohen Kappa statistics, where κ = (p(a)—p(e))/(1-p(e), where p(a) is the observed agreement, and p(e) is the probability of chance agreement. If there is complete agreement then κ = 1, while is there is no agreement other than that expected by chance, κ = 0.

For analysis of KRAS Ct values, which are not normally distributed, between the two methods we have quoted median values and the range. Statistical comparison was performed by paired non-parametric statistics (Wilcoxon rank sum test) using SPSS, ver 22 (IBM).

## Results

In order to compare two PCR based mutation detection methods, a total of 99 colorectal cancer samples were tested for *KRAS* and/or *BRAF* status by castPCR and Therascreen. Tables 1 and 2 summarise the genotyping results for both methods, while Table 3 shows the detailed results for each tumor tested. Both methods gave results in all patients, with no failed assays. Of the tumors included, 37 harboured *KRAS* mutation confirmed by both methods with re-testing of discrepant results (37%) and 13 *BRAF* p.V600E mutation by castPCR (13%). *BRAF* V600E mutation was not included in the Therascreen KRAS kit used. The most frequently detected *KRAS* alterations were codon 12 missense mutations c35G>T, p.G12V and c35G>A, p.G12D (both in 10 samples). Codon 13 mutations were also detected but at a lower rate (6 samples).

**Table 2:** A concordance matrix for *KRAS* for mutations included in both the Therascreen and castPCR assays.

KRAS only	castPCR	
	WT	Mutant
WT	61	1
Mutant	2	37

**Table 3:** Results for castPCR and Therascreen for each case included in the study.

**No.**	**TheraScreen Mutation Ct**	**TheraScreen DeltaCt**	**Therascreen Mutation**	**castPCR Mutation Ct**	**castPCR Delta Ct**	**castPCR Mutation**
P1	N/A	N/A	WT	N/A	N/A	WT
P2	N/A	N/A	WT	33.5	2.5	BRAF c.1799T>A
P3	28.4	0.5	**KRAS c.35G>C**	N/A	N/A	**WT**
P4	31.9	0.65	KRAS c.35G>T	33.1	3.0	KRAS c.35G>T
P5	32.1	0.8	KRAS c.35G>T	35.0	4.3	KRAS c.35G>T
P6	30.7	1.35	KRAS c.35G>T	28.0	2.3	KRAS c.35G>T
P7	34.6	3.69	KRAS c.35G>A	32.5	3.5	KRAS c.35G>A
P8	N/A	N/A	WT	N/A	N/A	WT
P9	N/A	N/A	WT	N/A	N/A	WT
P10	N/A	N/A	WT	26.2	0.6	BRAF c.1799T>A
P11	34	4.12	KRAS c.38G>A	34.7, 35.3	5.4, 9.0	KRAS c.38G>A, KRAS c.182A>G
P12	31.5	3.25	KRAS c.34G>T	28.0	3.1	KRAS c.34G>T
P13	N/A	N/A	WT	N/A	N/A	WT
P14	N/A	N/A	WT	N/A	N/A	WT
P15	N/A	N/A	WT	N/A	N/A	WT
P16	32.3	4.04	KRAS c.38G>A	29.3	4.0	KRAS c.38G>A
P17	35.1	1.02	KRAS c.35G>T	33.5	1.8	KRAS c.35G>T
P18	N/A	N/A	WT	N/A	N/A	WT
P19	29.1	1.85	KRAS c.35G>T	27.1	2.8	KRAS c.35G>T
P20	N/A	N/A	WT	N/A	N/A	WT
P21	32.1	2.91	KRAS c.34G>T	31.3	4.2	KRAS c.34G>T
P22	33.3	3.59	KRAS c.35G>A	33.0	3.9	KRAS c.35G>A
P23	35.9	4.14	KRAS c.38G>A	33.2	3.1	KRAS c.38G>A
P24	N/A	N/A	WT	N/A	N/A	WT
P25	36.7	7.58	**KRAS c.35G>C**	N/A	N/A	**WT**
P26	N/A	N/A	WT	N/A	N/A	WT
P27	N/A	N/A	WT	N/A	N/A	WT
P28	N/A	N/A	WT	N/A	N/A	WT
P29	34	2.75	KRAS c.35G>A	33.4	3.6	KRAS c.35G>A
P30	N/A	N/A	WT	N/A	N/A	WT
P31	37.6	3.87	KRAS c.38G>A	33.0	4.9	KRAS c.38G>A
P32	34.6	3.06	KRAS c.35G>A	38.5	7.7	KRAS c.35G>A
P33	N/A	N/A	WT	27.4	1.1	BRAF c.1799T>A
P34	N/A	N/A	WT	N/A	N/A	WT
P35	31.6	3.88	KRAS c.34G>A	29.5	2.3	KRAS c.34G>A
P36	N/A	N/A	WT	37.3	8.6	BRAF c.1799T>A
P37	N/A	N/A	WT	29.0	2.0	BRAF c.1799T>A
P38	N/A	N/A	WT	N/A	N/A	WT
P39	N/A	N/A	WT	36.0	9.4	BRAF c.1799T>A
P40	N/A	N/A	WT	N/A	N/A	WT
P41	N/A	N/A	WT	N/A	N/A	WT
P42	N/A	N/A	WT	N/A	N/A	WT
P43	33.2	3.56	KRAS c.38G>A	36.9	6.5	KRAS c.38G>A
P44	N/A	N/A	WT	N/A	N/A	WT
P45	28.9	1.1	KRAS c.35G>T	28.7	2.2	KRAS c.35G>T
P46	N/A	N/A	WT	N/A	N/A	WT
P47	32.7	6.38	KRAS c.34G>T	30.4	4.2	KRAS c.34G>T
P48	N/A	N/A	WT	N/A	N/A	WT
P49	32.4	6.97	KRAS c.35G>A	28.2	2.7	KRAS c.35G>A
P50	N/A	N/A	WT	N/A	N/A	WT
P51	N/A	N/A	WT	N/A	N/A	WT
P52	N/A	N/A	**WT**	39.4	9.4	**KRAS c.34G>A**
P53	32	4.35	KRAS c.35G>A	30.9	3.6	KRAS c.35G>A
P54	N/A	N/A	WT	N/A	N/A	WT
P55	N/A	N/A	WT	N/A	N/A	WT
P56	31.4	3.21	KRAS c.34G>T	30.4	3.7	KRAS c.34G>T
P57	N/A	N/A	WT	N/A	N/A	WT
P58	32.5	4.04	KRAS c.35G>A	32.8	4.3	KRAS c.35G>A
P59	30	3.12	KRAS c.35G>T	29.6	4.4	KRAS c.35G>T
P60	N/A	N/A	WT	N/A	N/A	WT
P61	N/A	N/A	WT	N/A	N/A	WT
P62	30.1	2.73	KRAS c.34G>C	27.7,	3.89,	KRAS c.34G>C
P63	N/A	N/A	WT	N/A	N/A	WT
P64	N/A	N/A	WT	N/A	N/A	WT
P65	N/A	N/A	WT	N/A	N/A	WT
P66	N/A	N/A	WT	27.3	1.6	BRAF c.1799T>A
P67	31.6	3.97	KRAS c.35G>A	28.6	3.6	KRAS c.35G>A
P68	32.2	4.26	KRAS c.35G>A	30.2	4.4	KRAS c.35G>A
P69	N/A	N/A	WT	N/A	N/A	WT
P70	N/A	N/A	WT	N/A	N/A	WT
P71	N/A	N/A	WT	26.9	1.4	BRAF c.1799T>A
P72	N/A	N/A	WT	N/A	N/A	WT
P73	N/A	N/A	WT	N/A	N/A	WT
P74	29.5	2.16	KRAS c.34G>C	28.1	3.2	KRAS c.34G>C
P75	29.7	1.68	KRAS c.35G>T	28.5	2.5	KRAS c.35G>T
P76	30.5	2.02	KRAS c.35G>T	29.8	4.5	KRAS c.35G>T
P77	N/A	N/A	WT	N/A	N/A	WT
P78	N/A	N/A	WT	N/A	N/A	WT
P79	N/A	N/A	WT	N/A	N/A	WT
P80	N/A	N/A	WT	N/A	N/A	WT
P81	34.1	2.33	KRAS c.34G>C	33.1	4.0	KRAS c.34G>C
P82	N/A	N/A	WT	N/A	N/A	WT
P83	N/A	N/A	WT	N/A	N/A	WT
P84	N/A	N/A	WT	27.1	1.6	BRAF c.1799T>A
P85	36.6	4.58	KRAS c.38G>A	34.3	5.1	KRAS c.38G>A
	N/A	N/A	WT	28.8	2.7	BRAF c.1799T>A
P86	30.8	2.3	KRAS c.35G>T	30.0, 35.1	4.1, 8.9	KRAS c.35G>T, **BRAF c.1799T>A**
P87	N/A	N/A	WT	N/A	N/A	WT
P88	N/A	N/A	WT	N/A	N/A	WT
P89	N/A	N/A	WT	N/A	N/A	WT
	N/A	N/A	WT	N/A	N/A	WT
P90	N/A	N/A	WT	27.0	1.5	BRAF c.1799T>A
W1	27.85	2.14	KRAS c.35G>C	30.5	3.4	KRAS c.35G>C
W2	30.53	2.26	KRAS c.35G>A	29.7	2.6	KRAS c.35G>A
W3	28.57	2.86	KRAS c.34G>T	31.4	4.0	KRAS c.34G>T
W4	N/A	N/A	WT	N/A	N/A	WT
W5	N/A	N/A	WT	28.7	1.3	BRAF c.1799T>A
W7	N/A	N/A	WT	N/A	N/A	WT
W8	N/A	N/A	WT	N/A	N/A	WT

Ct values for each reference gene are given with the ΔCt. An analysis matrix is used to determine the assay result (mutant or wild-type) according to thresholds defined by the manufacturer. There were three discrepant results (shown in bold): P3 showed *KRAS* c.35G>C by both methods on retesting, while P25 and P52 were both wild-type. Two cases (P11 and P86) showed amplification for more than one mutation. N/A, not applicable; WT, wild-type.

One sample harbored a *KRAS* codon 61 mutation (*KRAS* c.182A>G) as well as a *KRAS* p.G13D, KRAS c.38G>A mutation (Table 3, case P11). *KRAS* p.G12V and *BRAF* p.V600E mutations occurred together in a further tumor sample. Therascreen identified 37/38 *KRAS* mutations found by castPCR, though this is explicable on the basis of a mutation (*KRAS* c.182A>G) not present in Therascreen.

There were three discordant results between the two assay types on initial testing, with complete concordance from initial testing in 96/99 tumors (96%). This equates to κ = 0.94, showing excellent agreement. The number of *KRAS* wild-type (therascreen assay) patients who were also identified as *KRAS* wild-type by the castPCR was 61/62, giving a primary measure of concordance (p) of 0.984. Statistical sensitivity and specificity were 95% and 98% respectively.

Discordant tumors were then re-tested (Tables 1 and 3). One sample was negative in Therascreen and borderline positive (∆Ct = 9.41) by castPCR, however on re-testing it was considered wild-type in both assays. One tumor was called mutant initially by Therascreen and wild-type by castPCR, but was wild-type on re-testing by both assays. The third sample was initially tested mutant in Therascreen and borderline wild-type in castPCR but subsequent re-testing showed mutations with both methods.

The median mutation detection Ct values for *KRAS* mutations detected by both methods were 32.1 (range 27.9–37.6) for Therascreen and 30.4 (range 27.1–38.5) for castPCR (Wilcoxon p <0.012). The deltaCt values were 3.09 (range 0.65–6.97) and 3.79 (range 1.75–7.65) for Therascreen and castPCR respectively (Wilcoxon p <0.002). The castPCR Ct values for KRAS were therefore 2 cycles lower than Therascreen.

## Discussion

This study evaluates two PCR based assays for *KRAS* mutation detection in formalin-fixed paraffin-embedded CRC tissue samples. We observed three discrepancies between two mutation detection methods, which were solved by re-testing. Discrepancies are to be expected as most methods use cutoff values determined by relation to controls and have differing PCR efficiency. In tumors with little mutant DNA present, this will lead to differences in mutation detection. Similar small differences between methods using Therascreen as a comparator have been reported by others [18, 24–29]. Chang *et al*. [23] conducted a retrospective study of the CRYSTAL trial samples with Therasceen and LNA, showing excellent concordance, while Gonzalez de Castro *et al*. [18] showed similarly good concordance of 98% between Therascreen and cobas (Roche). Altimari et al. [27] showed reasonable concordance between Therascreen, pyrosequencing and Roche 454 sequencing, but noted the poor diagnostic sensitivity of Sanger sequencing. Therascreen has also been used to validate other PCR assays [25, 28], including high resolution melt analysis [29]. It should be noted that the diagnostic sensitivity of sequencing methods is dependent on the size of PCR products: FFPE tissue samples have fragmentation of their DNA and products > 150 bp suffer loss of diagnostic sensitivity [30].

In addition, it is increasingly accepted that intra-tumoral heterogeneity [31] can lead to the dilution of small proportions of mutant DNA by wild-type DNA despite high neoplastic cell numbers within the sample. It is unclear what proportion of mutation-containing neoplastic cells is required to produce clinical resistance: such considerations require very large datasets, which are not available. Although not a recommended by the manufacturers of either test, it is our practice to re-test results using further punches from another block when the Ct values are within one cycle of the threshold.

*KRAS* mutations were detected in 38% of CRC samples, which is in agreement with the frequency observed in previous reports [32, 33]. Interestingly, one tumor sample harbored two different *KRAS* mutations (p.G13D and p.Q61R) whilst another tumor was *KRAS*/*BRAF* double mutant. Both results probably reflect clonality within a tumor sample, or a collision tumor derived from two different initiating adenomas. Genetic heterogeneity within tumor samples is becoming increasingly evident as diagnostic tests with high sensitivity are able to detect subclones in tumor that have acquired additional mutations over time. Double mutations in *KRAS* in colorectal cancer have been reported before, however their clinical relevance is not known [34, 35]. In addition, it is becoming clear that not all *KRAS* mutations are equal: data are emerging to suggest that patients harboring the codon 13 c.38 G > A mutation may actually benefit from anti-EGFR treatment, suggesting incomplete activation of KRAS by this mutation [12, 36]. The effect of rare *KRAS*-activating mutations in codon 61 and 146 is also a matter of debate.

An oncogenic missense mutation p.V600E in *BRAF*, a downstream signalling molecule of *KRAS*, has been identified in around 5% of colorectal cancer tumors, though somewhat higher in this series at 10%, and results in activation of the MAPK signalling pathway [14]. *BRAF* p.V600E missense mutation is associated with poor prognosis in colorectal cancer and according to some recent reports, it has negative predictive value in anti-EGFR antibody therapy [18], though this has yet to reach sufficient levels of evidence to update clinical guidance [9–11, 37]. Thus it is likely that combined testing for *KRAS* mutations and *BRAF* p.V600E in CRC will be required for clinical practice in the near future [37]. Mutations in other genes, such as *PIK3CA* and *NRAS* may also influence anti-EGFR treatment efficacy in colorectal cancer [37]. This has recently led to the requirement that laboratories should test for *NRAS* as well as *KRAS* mutations with amended product labels for anti-EGFR antibody therapy [38].

Advantages of the custom castPCR plates used here is that they include Q61 *KRAS* mutation and also provide potentially valuable information on *BRAF* status in CRC samples. These can be further customised to suit the needs of individual laboratories. It is feasible to include these and other mutation hotspots in user customised castPCR plates, allowing larger numbers of clinically relevant mutations to be included and providing a real alternative to next generation sequencing, particularly for laboratories without this facility.

In conclusion, a good correlation was observed between the two methods. CastPCR shows slightly lower Ct values than Therascreen, however this is unlikely to be clinically significant. Our results show that castPCR is a reproducible and reliable assay that can be used as a diagnostic test for KRAS genotyping in formalin-fixed paraffin-embedded colorectal cancer samples.
